# Modeling trajectories of perceived leg exertion during maximal cycle ergometer exercise in children and adolescents

**DOI:** 10.1186/1471-2288-14-4

**Published:** 2014-01-09

**Authors:** Marianne Huebner, Zhen Zhang, Terry Therneau, Patrick McGrath, Paolo Pianosi

**Affiliations:** 1 Department of Statistics and Probability, Michigan State University, East Lansing, MI, USA; 2 Department of Health Sciences Research, Mayo Clinic, Rochester, MN, USA; 3 Departments of Psychology, Pediatrics, and Psychiatry, Dalhousie University, Halifax, NS, B3H 4J1, Canada; 4 Department of Pediatric and Adolescent Medicine, Mayo Clinic, Rochester, MN, USA

**Keywords:** Children, Perceived leg exertion, Borg scale, Ergometer exercise, Delay, Power model

## Abstract

**Background:**

Borg developed scales for rating pain and perceived exertion in adults that have also been used in pediatric populations. Models describing functional relationships between perceived exertion and work capacity have not been studied in children. We compared different models and their fits to individual trajectories and assessed the variability in these trajectories.

**Methods:**

Ratings of perceived exertion (RPE) were collected from 79 children. Progressive cycle ergonometric testing was performed to maximal work capacity with test duration ranging from 6‐ 12 minutes. Ratings were obtained during each 1‐minute increment. Work was normalized to individual maximal work capacity (Wmax). A delay was defined as the fraction of Wmax at which point an increase in ratings of leg fatigue occurred. Such a delay term allows the characterization of trajectories for children whose ratings were initially constant with increasing work. Two models were considered, a delay model and a power model that is commonly used to analyze Borg ratings. Individual model fit was assessed with root mean squared error (RMSE). Functional clustering algorithms were used to identify patterns.

**Results:**

Leg tiredness developed quickly for some children while for others there was a delay before an in‐ creased ratings of leg exertion occurred with increasing work. Models for individual trajectories with the smallest RMSE included a delay and a quadratic term (quadratic‐delay model), or a power function and a delay term (power‐delay model) compared to a simple power function. The median delay was 40% Wmax (interquartile range (IQR): 26‐49%) in a quadratic‐delay model, while the median exponent was 1.03 (IQR: 0.83‐1.78) in a power‐delay model. Nine clusters were identified showing linear or quadratic patterns with or without a delay. Cluster membership did not depend on age, gender or diagnosis.

**Conclusion:**

Children and adolescents vary widely in their capacity to rate their perceptions and exhibit different functional relationships between ratings of perceived exertion and work capacity normalized across individuals. Models including a delay term, a linear component, or a power function can describe these individual trajectories of perceived leg exertion during incremental exercise to voluntary exhaustion.

## Background

Ratings of perceived exertion (RPE) have been used to study physical activity and exercise tolerance in adults and children. Borg developed scales for rating pain and perceived exertion in adults [1] which has been employed in different exercises and different ages including children [2]‐[5]. This scale uses descriptive adjectives such as moderate and severe, for numbers from 0 to 10. Research studies have assessed whether children have the ability of gradation during exercise as this may depend on cognitive ability [6]. Low test‐to‐test variability in children using the Borg and OMNI scales has been reported by Pfeiffer et al. [2] in 57 adolescent athletic girls and by Lamb [7] in 70 preadolescent children. Mahon et al. [4] also noted consistency between trials in children ages 8‐11. The modified Borg scale was found to be adequate in 49 children with cystic fibrosis to determine exercise tolerance [3]. Some of these studies collected ratings at one time point, for example after a 6 minute walk test, or considered a test‐retest design to determine the consistency and reliability of ratings.

Individuals may rate perceived exertion differently at the same relative or absolute exercise intensity. To compare sensations generated by a particular stimulus such as exercise across individuals Borg developed a range‐model, reasoning that individuals will experience similar degrees of perceived exertion at their respective minima and maxima, providing anchors or calibration for rating sensation across individuals [8].

Adult studies demonstrated that subject‐estimated perceived exertion conformed to a function of the type

$$R=a+bW^{d}$$

where *d* is the exponent, *a* is the intercept at zero stimulus, and the coefficient *b* describes the speed of growth. Exponents averaged 1.7 for adults [9] or 1.1 [10]. Modeling of functional relationships between ratings of perceived exertion and work has not been done for pediatric subjects.

The goal of this study was to evaluate models to fit ratings of perceived leg exertion on a Borg scale as a function of maximum work capacity for individual children and adolescents and define models with parameters that are interpretable for the assessment of these individual patterns. We considered four models, a power model as described above, a linear model with a delay, and extension of these two models when introducing a delay in the power model *R*=*a*+*b*(*W*−*c*)^*d*^[9], and a delay model with a linear and a quadratic term.

## Methods

### Participants

Children with cardiopulmonary disease attending outpatient clinics at IWK Health Centre in Halifax, Canada were recruited to the study. Healthy control children were recruited from friends and relatives of hospital personnel, or siblings of these patients. Data were collected on 100 pediatric subjects with 4 to 12 ratings per subject. For reliable parameter estimation the data were limited to 79 children with at least 6 data points. The study was approved by the Research Ethics Board of the IWK Health Centre. Assent was obtained from all participants. Mature minors, or parents of younger children, signed informed consent.

### Procedure

Subjects performed continuous, graded, maximal, cycle ergometer (WE Collins) test exercise employing step increments of either, 50, 100, or 150 kpm per minute depending on size and age. Increments were chosen to achieve test duration of 6‐10 minutes ‐ until voluntary, symptom‐limited, exhaustion occurred as previously described [6]. Borg scale ratings were obtained during each 1‐minute increment. Work was normalized across subjects by expressing it as fraction of individual maximum work capacity (Wmax).

Both dyspnea and perceived leg exertion were measured, but we report only Borg ratings for perceived leg exertion since our principal aim was modeling the stimulus‐response function. We employed the Dalhousie pictorial scales [11] and the Borg scale [12], chosen because it had been used in previous investigations in adults with similar aims [13,14]. Subjects were first given an explanation of the scale by the research assistant.

The Borg scale of perceived exertion ranges from 0 to 10 with verbal descriptors 0= nothing at all, 0.5=very,very slight, 1=very slight, 2=slight, 3=moderate, 4=somewhat severe, 5=severe, 7=very severe, 9=very, very severe, 10=maximal [12,15]. The scale was mounted on a large clipboard in front of the participant, and had a sliding cursor located on the left margin. The cursor was moved manually from the top downward by the research assistant until it pointed to the rating that best described the subject’s degree of leg fatigue, at which point he or she activated a bell mounted on the handlebars.

### Models

Perceived leg exertion of individual children were modeled in terms of fraction of maximum work capacity. Two models have been proposed in the literature, the power model (P) which has been used for fitting individual curves in adults [10], and the power delay model (PD) [9] which has not been used for fitting study data to the best of our knowledge. Since an increase in leg fatigue was observed after some delay, a lag or delay model (D) was developed for this study. A “lag” or delay was defined as % Wmax at which point a clear increase in ratings of leg fatigue occurred. While the power model accounted for curvature of the model fits, but not for a delay, and the delay model accounted for a delay, but not a curvature, we also considered a quadratic‐delay model (QD) and a power model with delay (PD). The quadratic delay model includes an intercept, a linear term, and a quadratic term. This allows for flexibility that the observed pattern may be linear without a curvature. The parameters in each model were estimated using a quasi‐Newton method with box constraints, where a variable can be given a lower bound [16]. For the purpose of estimating the parameters constraints were introduced, namely the delay is constrained to be larger than the initial observed % maximum work capacity, the exponent must be positive. Since the ratings increase, and a minimum proportion of work capacity must be observed, these are reasonable assumptions. The four models were defined as follows. For each child (*i*=1,…,*n*) with *j*=1,…,*n*_*i*_ observations, leg exertion (*Y*=(*y*_*ij*_)) is modeled as a function of % maximum work capacity (*X*=(*x*_*ij*_)).

$$\begin{array}{lll} \;\text{Power model (P)}:y_{\text{ij}} & =a_{i}+b_{2i}x_{\text{ij}}^{d_{i}}+\varepsilon_{\text{ij}}\; & \;\\ \;\text{Delay model (D)}:y_{\text{ij}} & =a_{i}+b_{1i}(x_{\text{ij}}-c_{i}\vee 0)+\varepsilon_{\text{ij}}\; & \;\\ \;\text{Power‐delay model (PD)}:y_{\text{ij}} & =a_{i}+b_{2i}{\left(x_{\text{ij}}\;-\;c_{i}\vee 0\;\right)}^{d_{i}}\;+\;\varepsilon_{\text{ij}}\; & \;\\ \;\text{Quadratic‐delay model (QD)}:y_{\text{ij}} & =a_{i}+b_{1i}(x_{\text{ij}}-c_{i}\vee \;0)\; & \;\\ & \;+b_{2i}{\left(x_{\text{ij}}-c_{i}\vee \;0\right)}^{2}+\varepsilon_{\text{ij}}\; & \; \end{array}$$

where $a_{i},b_{1i},b_{2i},d_{i}\in R,d_{i}\geq 0,$ and *c*_*i*_> min*j*(*x*_*ij*_). For all models it is assumed that $\varepsilon_{\text{ij}}∼N(0,\sigma_{i}^{2})$.

#### Model assessment

**Figure 1:**
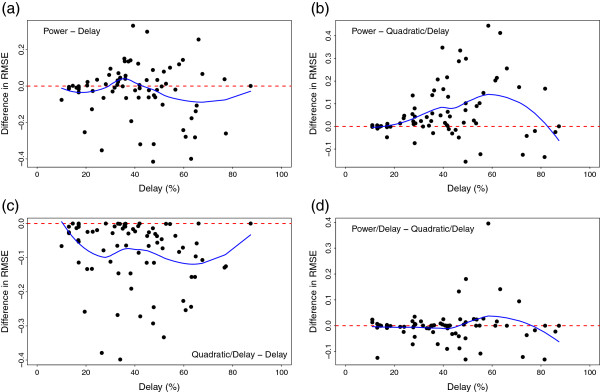
**Difference in RMSE for different models compared to size of delay.** The difference in RMSE of model 1 minus RMSE of model 2 for individual curves are plotted against the estimated delay (n=79 points) and overlaid with a loess smoothing curve. **(a)** RMSE Power model model minus RMSE Delay model, **(b)** RMSE Power model minus RMSE Quadratic delay model, **(c)** RMSE Quadratic delay model minus RMSE Delay model, **(d)** RMSE Power delay model minus RMSE Quadratic delay model.

**Figure 2:**
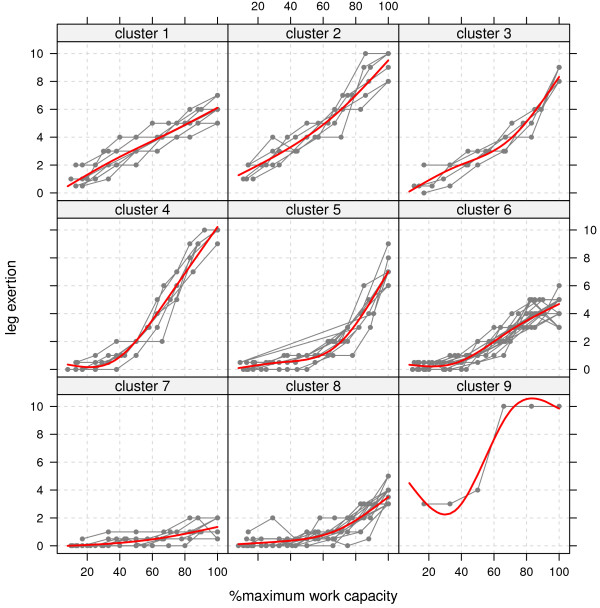
**Functional clusters of individual curves.** Individual curves (n=79) are clustered by their trajectories using a functional clustering model (see Additional file 1). Some panels show steep or slow linear growth, quadratic growth or curves with lag, and are also distinguished by the maximum rating before the end of the exercise.

For model assessment we calculated root‐mean‐square error RMSE${}_{i}=\sqrt{\overset{n_{i}}{\underset{j=1}{\sum }}{\left(y_{\text{ij}}-{ŷ}_{\text{ij}}\right)}^{2}/n_{i}}$. In place of the Akaike information criterion (AIC) we used the corrected AIC (AICc), which imposes a penalty on the number of parameters for finite or sparse samples. The Bayesian information criterion (BIC) was also calculated. These quantities are defined as

$$\begin{array}{lll} \;{\text{AIC}}_{i} & =2k-\log(L_{i})\; & \;\\ \;{\text{AICc}}_{i} & ={\text{AIC}}_{i}+\frac{2k(k+1)}{n_{i}-k-1}\; & \;\\ \;{\text{BIC}}_{i} & =k\log n_{i}-n_{i}\log(L_{i})\; & \; \end{array}$$

where *L*_*i*_ is the maximum of the individual likelihood function. The number of coefficients are *k*=3,3,4 and 4 for the four models. For the purpose of RMSE comparisons (Figure 1) one subject with ratings at only two levels was removed (cluster 9 in Figure 2).

#### Mixed effects models

Nonlinear mixed effects models can be used to study average parameters for delay and growth. The models are similar to the PD and QD models but with additional terms to model overall variation and individual variation (MPD or MQD). This assumes that the delay is constant across all individuals. More general mixed effects models include the delay as a random effect, a mixed effects quadratic‐delay model with varying delays for each subject (MPDV or MQDV).

$$\begin{array}{lll} \;\text{MQD}:y_{\text{ij}} & =a+b_{1}(x_{\text{ij}}\;-\;c\vee \;0)+b_{2}{\left(x_{\text{ij}}-c\vee \;0\right)}^{2}+u_{i}+\varepsilon_{\text{ij}},\; & \;\\ \;\text{MPD}:y_{\text{ij}} & =a+b_{2}{\left(x_{\text{ij}}\;-\;c\vee \;0\right)}^{d}+u_{i}+\varepsilon_{\text{ij}}\; & \;\\ \;\text{MQDV}:y_{\text{ij}} & =a+b_{1}(x_{\text{ij}}\;-\;c_{i}\vee \;0)+b_{2}{\left(x_{\text{ij}}-c_{i}\vee \;0\right)}^{2}\;+\;u_{i}+\varepsilon_{\text{ij}}\; & \;\\ \;\text{MPDV}:y_{\text{ij}} & =a+b_{2}{\left(x_{\text{ij}}\;-\;c_{i}\vee \;0\right)}^{d}+u_{i}+\varepsilon_{\text{ij}}\; & \; \end{array}$$

where $a,b_{1},b_{2},d\in R$, and *c*∈[ min*i*,*j*(*x*_*ij*_), max*i*,*j*(*x*_*ij*_)] for MQD and MPD, or *c*_*i*_=*c*+*γ*_*i*_, *c*_*i*_∈[ min*j*(*x*_*ij*_), max*j*(*x*_*ij*_)] for MQDV and MPDV.

The overall variation is modeled by $\epsilon ∼N(0,\sigma^{2})$, and the random intercept by $u_{i}∼N(0,\tau^{2})$. For MPDV and MQDV the random delay is assumed to follow a normal distribution $\gamma_{i}∼N(0,\nu^{2})$. All random components are assumed to be independent. Parameters are estimated using a Bayesian approach by choosing conjugate priors for *a*,*b*_1_,*b*_2_,*σ*^2^,*τ*^2^, and non‐informative uniform priors for the delay *c* and the exponent *d* (see Additional file 1). A dispersed prior density was chosen for the distribution of the parameters to allow for more data‐driven estimators.

These four competing models were implemented, the mixed effects quadratic‐delay model, the mixed effects power‐delay model, each with common delay, and both models with delay as random effects. Under each implementation three Monte Carlo Markov Chains were run. Convergence was well achieved after 10,000 iterations, namely when Potential Scale Reduction Factor $\sqrt{R}<1.2$ for all parameters [17]. Further 1,000 iterations were sampled every 10 iterations to obtain a total of 300 posterior samples for all parameters and random effects. For model comparisons the Deviance Information Criterion (DIC) for mixed‐effects models based on the complete likelihood was used [18]. A smaller DIC indicates a better predictive power of the model.

### Computing environment

For estimating the model parameters of models P, D, PD, QD the function *optim* was used in the statistical software R 2.15.3 [19]. The nonlinear mixed effects models were fitted using MATLAB R2013a (Mathworks Inc, Natick Massachusetts).

## Results

Ratings of Borg scale for leg exertion were collected from 79 children: 32 healthy, 26 asthma, 21 cystic fibrosis children. The median age was 12 (range 8‐18), and 58% were boys. Characteristics of the participants are described in Table 1.

**Table 1:** Characteristics and maximal exercise data (means ± SD) of children who performed progressive bicycle exercise

**Characteristic**	**Controls**	**Cystic fibrosis**	**Asthma**
Boys:Girls (n)	17:15	15:6	14:12
Age (years)	12.4 ± 2.5	14.1 ± 2.3	12.0 ± 2.8
Height (cm)	155 ± 13	158 ± 11.6	150 ± 14
Weight (kg)	51.9 ± 15.9	48.7 ± 11.1	45.8 ± 16.4
FEV1 (% predicted)	103 ± 17	75 ± 18	96 ± 16
$\dot{V}O_{2}$ (ml /min · kg)	36.0 ± 10.1	28.7 ± 8.3	33.4 ± 8.7
HR (bpm)	193 ± 9	185 ± 15	189 ± 11
${\dot{V}}_{E}$ (l/min)	77.8 ± 22.3	77.8 ± 18.1	77.6 ± 24.0

*Abbreviations:*$\dot{V}O_{2}$ oxygen uptake, ${\dot{V}}_{E}$ minute volume, *FEV1* forced expiratory volume, *HR* heart rate.

### Model fit of individual curves

Both PD and QD models can represent linear growth or quadratic growth in ratings. The delay was estimated for each subject. Considering whether the size of the delay influences the RMSE in the models we plotted the difference in RMSE for the two models and overlaid it with a loess smoother (Figure 1). In this figure it is clear that the power‐delay model and the quadratic‐delay model have similar root mean square error for longer and shorter delays. A difference between the simpler power model and delay model could not be detected, although it is possible that the delay model may have an advantage over the power model for longer delays. Models incorporating delay and curvature had smaller RMSE for all sizes of delay in individual curves (Figure 1, P vs QD or QD vs D). The model fit for the power model and delay model were similar with average root mean square error of 0.30 and 0.33, respectively and BIC of 9.72 and 10.17, respectively (Table 2). Adding a delay to the power model and a quadratic terms to the delay model reduced the RMSE to 0.23 and 0.25 and BIC 7.90 and 7.87, respectively (Table 2). Smaller BIC or smaller RMSE are indicators of a better fit. This large decrease in RMSE was likely due to “edge effects," namely these were individuals who reached their maximum rating, but then continued the exercise longer. This was the case when ratings increased sharply and then reached a plateau in which case the coefficient of the quadratic term would be negative, for example RPE =0.5+30.31∗ max(0,*w*−0.46)−22.35∗ max(0,*w*−0.46)^2^, in the QD model. From the estimated curve one would identify this subject as having a long delay, a sharp increase in ratings, and then a plateau. In the PD model plateaus correspond to an exponent less than 1, e.g. in this case RPE =0.4+16.7∗(*x*−0.40)^0.9^.

**Table 2:** Comparison of RMSE, AICc, and BIC for the models of perceived exertion, median (first, third quartiles)

**Model**	**RMSE**	**AICc**	**BIC**
Power	0.30 (0.17,0.47)	17.17 (11.0, 24.27)	9.72 (0.23, 16.44)
Delay	0.33 (0.22, 0.52)	19.39 (11.66, 24.27)	10.17 (4.39, 17.52)
Power with delay	0.23 (0.12, 0.42)	29.12 (18.81, 40.36)	7.90 (‐1.05, 16.55)
Quadratic with delay	0.25 (0.16, 0.41)	28.40 (18.32, 43.82)	7.87 (1.75, 16.50)

*Abbreviations: RMSE* root mean square error, *AIC* Akaike information criterion, *AICc* corrected AIC, *BIC* Bayesian information criterion.

The median coefficient of the quadratic term in the QD model is 0 (interquartile range (IQR): ‐4.34,5.80) (Table 3). This reflects the fact that a large number of individuals had plateaus at the maximum work capacity.

**Table 3:** Summary of estimated model parameters for models of perceived exertion: median (1st quartile, 3rd quartile)

**Parameter**	**P**	**D**	**PD**	**QD**
Intercept (*a*)	‐0.04 (‐0.29,0.32)	0.37 (0.00,0.54)	0.36 (0.00, 0.60)	0.39 (0.00, 0.62)
Slope 1(*b*_1_)		7.32 (5.92,10.52)		7.01 (3.18, 11.90)
Slope 2 (*b*_2_)	5.13 (3.45,7.25)		7.00 (5.45,10.67)	0.00 (‐4.34,5.80)
Exponent (*d*)	2.03 (1.40,2.92)		1.03 (0.83,1.78)	
Delay (*c*)		0.38 (0.27,0.50)	0.30 (0.17, 0.41)	0.40 (0.26,0.49)

*Abbreviations:P* power model, *D* delay model, *PD* power model with delay, *QD* quadratic model with delay.

In the PD model individual growth curves with a long delay can have a large exponent and small coefficients to model the increase in ratings. For example, one subject had an estimated delay of 66 %Wmax before the ratings for leg fatigue increased rapidly. The corresponding power model was RPE = −0.1+4.2∗*w*^4.7^. The exponent in the corresponding power‐delay model was closer to 1, RPE = 20+11.8∗ max(0,*w*−0.66)^1^ and would result in a better fit. Estimated parameters in the models can be interpreted directly for individuals or need to be considered in conjunction with other model parameters. For example, the delay in the power delay model or the (D) or (QD) models is related to individual perceptions, while the exponent in the power model must be considered in connection with the coefficient of the power term.

### Estimated model parameters from pooled data

The median (IQR) of model parameters from the individual curves are reported in Table 3. The median exponent for the power model (P) was 2.03 (IQR 1.40‐2.92). However when a delay is added to the power model (PD), then the median exponent drops to 1.03 (IQR 0.83‐1.78). The median coefficient of the quadratic term in the QD model was 0.0 (IQR ‐4.34‐5.80). The delay in the delay model (D) and the quadratic delay model (QD) were estimated to be 38 and 40 %Wmax respectively. The median delay from the PD model is short at 30 %Wmax (IQR 17‐41 %), but a difference of 10% in Wmax is clinically not relevant in light to moderate exercise.

In the mixed effects models where the delay was assumed to be constant across all individuals, the estimated mean delay was 0.09 (9% Wmax) for both the MQD and MPD models (Table 4). Since the smallest fraction of maximum work capacity was 0.08, an estimated delay *c* that is close to its lower limit indicated a negligible delay effect. Thus pooled data masked the individual delay effect and the trajectories increased with respect to some power function. However when the delay was allowed to vary for individuals (MPDV, MQDV), the estimated average delay was similar for both models, 47 % Wmax (95% CI: 41, 59%). This was larger than the average delay from the individual PD and QD models due to the choice of uniform prior. The quadratic term had a positive coefficient, but the 95% confidence interval included zero for MQDV; the mean exponent for MPDV was 1.28 (95% CI: 0.40, 1.67) and included 1. The DIC was about 2100 for mixed effects models with common delay and about 1900 for models with varying delays, hence the fit was better for varying delay models.

**Table 4:** Summary of estimated model parameters for mixed effects models of perceived exertion: posterior mean (2.5%th quantile, 97.5%th quantile)

**Parameter**	**MPD**	**MQD**	**MPDV**	**MQDV**
Intercept(*a*)	0.36(−0.01,0.71)	0.32(−0.07,0.71)	0.71(0.40,1.14)	0.72(0.47,0.94)
Slope 1(*b*_1_)		0.66(−0.66,1.87)		5.24(3.31,11.12)
Slope 2(*b*_2_)	6.06(5.67,6.51)	5.49(4.24,6.79)	9.88(5.76,11.27)	5.76(−4.41,8.34)
Exponent(*d*)	1.85(1.59,2.13)		1.28(0.40,1.67)	
Delay(*c*)	0.09(0.08,0.12)	0.09(0.08,0.12)	0.47(0.41,0.59)	0.48(0.43,0.57)
*σ*^2^	1.30(1.15,1.48)	1.31(1.16,1.48)	0.96(0.68,1.80)	0.90(0.69,1.61)
*τ*^2^	1.77(1.27,2.51)	1.80(1.30,2.46)	0.41(0.17,1.12)	0.36(0.18,0.61)
*δ*^2^			0.09(0.03,0.35)	0.17(0.03,1.03)
DIC	2101.73	2102.41	1919.93	1914.91

*Abbreviations: MQD* mixed effects quadratic‐delay model, *MPD* mixed effects power‐delay model, *MQDV* mixed effects quadratic model with varying delay, *MQDV* mixed effects power model with varying delay, *DIC* Deviance Information Criterion.

The CPU time with a total 11,000 iterations on a 64‐bit, 8 GB RAM and Intel(R) Core(TM) i7‐3520M CPU 2.90 GHz laptop using MATLAB R2013a, were approximately 1.46 minutes for MQD, 17.19 minutes for MQDV, 48.57 minutes for MPD, and 100 minutes for MPDV.

### Functional clusters

Large differences and patterns for children’s responses exists. Trajectories identified by functional clustering algorithm are shown in Figure 2. Clusters 1, 2 and 3 show children with linear growth in ratings, clusters 4, 5, and 6 identified subgroups with delay and a power function, clusters 6, 7 and 8 represent groups with little increase. This illustrates the need for estimating individual growth functions in children. At maximum work capacity not all children perceived exertion to the same degree, some have ratings less than 6 while others rate their exertion at maximum possible rating. There was no clear distinction between boys and girls, age, or diagnosis (Table 5), although a larger proportion of children younger than 13 belonged to clusters 7 and 8 with a lower maximum rating as noted below.

**Table 5:** Description of cluster membership by gender, age, and diagnosis

	**n**	**Male**	**%**	**Age < 13**	**%**	**Asthma**	**%**	**CF**	**%**	**Healthy**	**%**
Cluster 1	8	5	62.5	4	50.0	5	62.5	1	12.5	2	25.0
Cluster 2	6	3	50.0	1	16.7	3	50.0	1	16.7	2	33.3
Cluster 3	5	3	60.0	0	0.0	1	20.0	4	80.0	0	0.0
Cluster 4	6	4	66.7	4	66.7	1	16.7	2	33.3	3	50.0
Cluster 5	9	4	44.4	3	33.3	2	22.2	3	33.3	4	44.4
Cluster 6	17	9	52.9	6	35.3	4	23.5	6	35.3	7	41.2
Cluster 7	10	6	60.0	8	80.0	5	50.0	1	10.0	4	40.0
Cluster 8	17	11	64.7	11	64.7	5	29.4	2	11.8	10	58.8
Cluster 9	1	1	100.0	0	0.0	0	0.0	1	100.0	0	0.0

*Abbreviations: CF* cystic fibrosis.

### Effects of age, gender, and diagnosis

When considering subgroups by gender or diagnosis the medians of the parameters were similar. In the power‐delay model the median exponent for girls was 1.06 vs 1.01 for boys with a delay of 0.26 for girls and 0.36 for boys. The exponent in the asthma group was 1.01 vs 1.03 for cystic fibrosis patients, and 1.04 for healthy controls, while the median delay for these groups was 24, 36, and 32 %Wmax, respectively.

Most children (89%) had a rating less than 10 (the maximum possible). The median rating at maximum work capacity was 5 (IQR 3.75‐7.5). Younger children, less than 13 years, had a lower maximal rating (median 4, IQR 3‐6) than older children (median 6, IQR 4‐8), p=0.023.

## Discussion

We evaluated models to fit individual patterns of perceived leg exertion as a function of exercise intensity expressed as proportion of Wmax. Four models were studied, a power model based on the Borg scale, a linear model with a delay, and extension of these two models when introducing a delay in the power model, and a delay model with a linear and a quadratic term. Our main findings were that models incorporating both delay and curvature had the smallest error term (RMSE) and were flexible enough to fit varying individual trajectories. Patterns in the individual trajectories can be illustrated by using functional clusters which identified linear and quadratic patterns, distinguished by the rate of rise in perceived leg exertion and the size of the delay as work became progressively harder.

Borg and Kaijser [10] also estimated individual curves for perceived exertion but in the case of adults. The mean exponent was 1.2 (SD 0.4), but closer to 1 when an intercept term was used. The individual curves for adults were more homogeneous and mostly linear and thus were comparable between adults. In contrast individual curves for children from our study varied widely and could exhibit a linear trend or rise as a power function with or without a delay.

A delay in the model arises when ratings of perceived exertion stay relatively constant before an estimated threshold at some percentage of Wmax. This may indicate a lack of ability in gradation or serialization for rating perceived exertion in our pediatric population. The intercept can be interpreted as a mathematical construct for a better fit or a threshold for a baseline or resting level of sensation. Both power‐delay and quadratic‐delay models are able to represent linear or quadratic growth in ratings. The exponent in the power model depends on the multiplicative constant to obtain a fit, so that large exponents are balanced with small coefficients for the power function. A quadratic‐delay model simplifies the interpretation, since it has a fixed exponent, and only the coefficients of the quadratic and linear terms need to be estimated. If this is coefficient is zero, then the curve is linear, non‐zero coefficients measure the strength of the curvature. It is also possible to use the quadratic delay model to estimate growth curves with an inflection, for example when ratings increase sharply and then level off. The computational burden for the quadratic delay model was much smaller than for the power‐delay model. In the mixed effects model with varying delays this amounted to 17 minutes versus 100 minutes CPU time. Otherwise the model fits for power‐delay model and quadratic‐delay models were comparable in terms of RMSE, AICc, BIC, DIC. Aggregated reporting in studies relating stimulus with response can obscure individual differences. The choice of mixed effects models have to be carefully considered and are more meaningful when estimating varying delays. However mixed effects models do not allow the estimation of individual curves. Borg and Kaijser [10], who opted for estimating individual curves in 20 adults, noted one individual with an exponent >2.8 in the respective power function. Individual differences were even more pronounced in our study in a pediatric population compared to differences in individual adult ratings in the aforementioned study. If individual growth functions are comparable as may be the case in adult populations, pooling data is a reasonable choice to estimate a generic function that could be used as a reference. In such cases a the estimated exponents in a power model can be interpretable assuming the coefficients of the power term is similar for individuals. However when individual growth functions exhibit different functional relationships and differ in their maximum work capacity, as was the case in our pediatric subjects, then curvature or delay may be lost in aggregate data when comparing average model parameters.

The median maximum rating for leg exertion among our pediatric subjects was only slightly greater than half the maximum possible value 10. Other investigators using different rating scales of perceived exertion also reported sub maximal ratings [7,13,14]. In this regard, it is worth noting that younger children had lower median rating (4, IQR 3‐6) than did children over 13 years of age (median 6, IQR 4‐8). Any explanation why more than half our subjects reported a delay in rating perceived exertion above baseline or resting values must be pure speculation. The period between 8‐12 years corresponds to a developmental level when children learn to differentiate sensations arising from differing parts or regions of their body [20]. Lamb noticed unreliability at lower exercise levels, which he postulated could be due to a number of factors such as motivation and perceptual development [7]. Some children’s ratings were flat with increasing work rather than a continuous rise, perhaps reflecting inability to understand the scale or properly gauge their perceived exertion, a phenomenon also noted by Swain et al. in a study in children ages 7‐11 years [21]. It has been suggested that children at this developmental stage (age range is a generalized approximation) must exercise at a relatively high intensity before they are able to accurately differentiate feelings arising in specific parts or regions of their body such as perceived leg exertion. Moreover, these same authors argued that children at this stage (age being a proxy measure) could distinguish up to four levels of exercise intensity during cycle ergometry [22]. Although it has been observed that adult males experience the same degree of exertion at work maximum [5], this was not the case for children in this study. An explanation could be that children and adolescents lack the antecedent experiences and required perceptual anchors needed to accurately gauge the greatest imaginable perceived leg exertion at maximal exercise. We also observed children with non‐monotone growth functions, where the ratings may decrease and then increase, typically in children with low ratings. However submaximal rating was also seen in 80% of 460 adults by Killian et al. [15] who explained this phenomenon by tolerance for discomfort.

A limitation for model fitting was the number of observations with as few as six and at most 12 in this sample of children and adolescents. We believe our subjects exercised to their respective maxima judging from peak exercise values shown in Table 1, but lower work capacity will set a limit on the number of graded measurements one can make in younger children such that this group had fewer measurements than seen in adults. There can be a risk of truncation that affects model fitting since the highest rating is 10. Another limitation is that the functional relationships were estimated from one trial. The level of habitual activity for each subject was not assessed. It would be useful to assess the children in a second trial to observe any changes in the functional relationship and increasing comfort with the process of rating.

## Conclusion

Children and adolescents have widely varying capacity to rate their perception(s) and exhibit different trajectories of perceived leg exertions during incremental exercise to voluntary exhaustion. These can nonetheless be described with model parameters for a delay in increase and the rate of increase (linear, quadratic, or some power exponent). Models with a delay term and curvature best describe the functional relationship between ratings of perceived exertion and work capacity normalized across individuals. Further research is needed to examine such functions in other pediatric populations.

## Abbreviations

RPE: Rating of perceived exertion; Wmax: Maximum work capacity; P: Power model; D: Delay model; PD: Power-delay model; QD: Quadratic-delay model; RMSE: Root mean square error.

## Competing interests

The authors declare that they have no competing interests.

## Authors’ contributions

MH, TT, and ZZ defined the models and interpreted the results. MH and ZZ performed the analyses and prepared the manuscript. PP and PM designed the study and collected the data, obtained research approval, and contributed to the writing of the manuscript. All authors read and approved the final manuscript.

## Pre-publication history

The pre-publication history for this paper can be accessed here:

http://www.biomedcentral.com/1471-2288/14/4/prepub

## Supplementary Material

Additional file 1 Mathematical supplement. Click here for file
