# Supplementary material for: Modeling trajectories of perceived leg exertion during maximal cycle ergometer exercise in children and adolescents
Source: BMC Med Res Methodol. 2014 Jan 9;14:4. doi: 10.1186/1471-2288-14-4 (PMC3910225; doi:10.1186/1471-2288-14-4)
Supplement: Additional file 1 — Mathematical supplement. [file 1471-2288-14-4-S1.pdf]

M. Huebner et al: Modeling trajectories of perceived leg exertion during maximal cycle ergometer exercise in children and adolescents

Mathematical Supplement

Functional clustering

In order to identify subgroups an exploratory functional clustering for sparse data was applied. The clustering model by James et al [1] assumes that the observed values y_i for individuals i can be constructed from a function g_i subject to measurement errors ε_i at measurements of %Wmax, $x_{i1}, \ldots, x_{in_i}$.

$$Y_i = g_i + \varepsilon_i, \quad i = 1, \ldots, n$$

The measurement errors have mean zero and are uncorrelated. It is assumed that unobserved x are missing at random. Spline basis functions are used for the functions g. This leads to a functional clustering model [1]

$$\begin{aligned} Y_i &= S_i(\lambda_0 + \Lambda\alpha_{z_i} + \gamma_i) + \varepsilon_i, \quad i = 1, \ldots, n \\ & \varepsilon_i \sim \mathcal{N}(0, \sigma^2 I), \gamma_i \sim \mathcal{N}(0, \Gamma) \end{aligned}$$

where $S_i = (s(x_{i1}), \ldots, s(x_{in_i}))$ is the spline basis matrix for the i-th individual. The model $\lambda_0 + \Lambda\alpha_{z_i}$ parameterizes the cluster means where z_i refers to the unknown cluster membership.

Nonlinear mixed effects models

For each child from $i = 1$ to $n = 79$, each with n_i observations we consider the Mixed-effects Quadratic-Delay model (MQD)

$$y_{ij} = a + b_1 (x_{ij} - c \vee 0) + b_2 (x_{ij} - c \vee 0)^2 + u_i + \epsilon_{ij}, \quad a, b_1, b_2 \in \mathbb{R}, c \in [\min_{i,j}(x_{ij}), \max_{i,j}(x_{ij})]$$

where $a \vee b$ denotes $\max\{a, b\}$. We assume $\epsilon \sim_{ind} \mathcal{N}(0, \sigma^2)$ with σ^2 measures the overall variation, and the random effect $u_i \sim_{ind} \mathcal{N}(0, \tau^2)$ with τ^2 measures the variation corresponding to each individual. Let $Y_i = (y_{i1}, y_{i2}, \cdots, y_{in_i})'$ with n_i observations for the ith subject and let $\boldsymbol{Y} = (Y_1', Y_2', \cdots, Y_n')'$. By stacking all $N = \sum_i n_i$ observations, the model can be written as:

$$\boldsymbol{Y} = \boldsymbol{X}(c)\boldsymbol{\beta} + \boldsymbol{Z}\boldsymbol{u} + \boldsymbol{\epsilon}$$

where $\boldsymbol{X}(c)$ is the design matrix with each row $(1,\, x_{ij} - c \vee 0,\, (x_{ij} - c \vee 0)^2)$, and $\boldsymbol{\beta} = (a, b_1, b_2)'$. The parameters to be estimated are $\boldsymbol{\theta} = \{a, b_1, b_2, c, \sigma^2, \tau^2\}$. A Bayesian approach is used by choosing conjugate priors for the slope $\boldsymbol{\beta} = (a, b_1, b_2)'$ and the variation components σ^2, τ^2, and a flat prior for the delay c over its finite support. Specifically,

$$\begin{aligned}
\pi(\boldsymbol{\beta}) &= \mathcal{N}_3(\mathbf{0}_3,\, \delta^2 I_3) \\
\pi(\sigma^2) &= \mathrm{IG}(a_\sigma, b_\sigma) \\
\pi(\tau^2) &= \mathrm{IG}(a_\tau, b_\tau) \\
\pi(c) &= \mathrm{Uniform}(\min_{i,j}(x_{ij}),\, \max_{i,j}(x_{ij}))
\end{aligned}$$

where $\mathcal{N}_p(\mu, \Sigma)$ denotes a p-dimensional Gaussian distribution with mean μ and covariance Σ, and $\mathrm{IG}(a, b)$ denotes Inverse Gamma distribution with shape parameter a and scale parameter b. In practice, we choose the hyper-parameters $\delta^2 = 10^3$, $a_\sigma = a_\tau = 2$ and $b_\sigma = b_\tau = 0.01$ which yields a dispersed prior density for less subjectivity. The full conditional distributions for Gibbs sampler are:

$$\begin{aligned}
\pi(\boldsymbol{\beta}|\cdots) &= \mathcal{N}_3(\mu_{\boldsymbol{\beta}}, \Sigma_{\boldsymbol{\beta}}) \quad \begin{cases} \Sigma_{\boldsymbol{\beta}} = (\sigma^{-2}\boldsymbol{X}(c)'\boldsymbol{X}(c) + \delta^{-2} I_3)^{-1} \\ \mu_{\boldsymbol{\beta}} = \sigma^{-2}\Sigma_{\boldsymbol{\beta}}\boldsymbol{X}(c)'\boldsymbol{Y} \end{cases} \\
\pi(\sigma^2\,|\cdots) &= \mathrm{IG}\left(N/2 + a_\sigma,\, \boldsymbol{\epsilon}'\boldsymbol{\epsilon}/2 + b_\sigma\right), \quad \text{where } \boldsymbol{\epsilon} = \boldsymbol{Y} - \boldsymbol{X}(c)\boldsymbol{\beta} - \boldsymbol{u} \\
\pi(\tau^2\,|\cdots) &= \mathrm{IG}\left(n/2 + a_\tau,\, \boldsymbol{u}'\boldsymbol{u}/2 + b_\tau\right) \\
\pi(c\,|\cdots) &\propto \exp\{-\frac{1}{2\sigma^2}\sum_{i,j}(\Delta_{ij} - u_i)^2\} \\
\pi(u_i\,|\cdots) &= \mathcal{N}_1\left(\frac{\tau^2}{n_i\tau^2 + \sigma^2}\sum_{j=1}^{n_i}\Delta_{ij},\, \frac{\tau^2}{n_i\tau^2\sigma^{-2} + 1}\right)
\end{aligned}$$

where $\Delta_{ij} = y_{ij} - a - b_1 (x_{ij} - c \vee 0) - b_2 (x_{ij} - c \vee 0)^2$. Note $\pi(c \mid \cdots)$ is not in closed form and can be computed numerically using Griddy-Gibbs sampler [2] over its finite support $[\min_{i,j}(x_{ij}),\, \max_{i,j}(x_{ij})]$. For instance, $\min_{i,j} x_{ij}$ is 0.08 for %maximum work capacity. An estimated delay c that is close to its lower limit indicates a negligible delay effect. This can happen due to pooled data masking delay effects for individuals.

Therefore we extend the mixed-effects quadratic-delay model to allow varying delay effects, by incorporating another random effect γ_i for the delay parameter c:

$$\begin{aligned} y_{ij} &= a + b_1 (x_{ij} - c_i \vee 0) + b_2 (x_{ij} - c_i \vee 0)^2 + u_i + \epsilon_{ij}, \quad a, b_1, b_2 \in \mathbb{R} \\ c_i &= c + \gamma_i, \quad c_i \in [\min_j(x_{ij}),\, \max_j(x_{ij})] \end{aligned}$$

with the constraint $\sum_i \gamma_i = 0$ to avoid identifiability issues. Again we assume $\epsilon \sim \mathcal{N}(0, \sigma^2)$ with σ^2 measures the overall variation, the random intercept $u_i \sim \mathcal{N}(0, \tau^2)$ and random delay $\gamma_i \sim \mathcal{N}(0, \nu^2)$ with τ^2 and ν^2 measure the variation of due to each individual. All three components are assumed to be independent. This Mixed-effects Quadratic-Delay Model with Varying delays (MQDV) can be written in matrix format

$$\boldsymbol{Y} = \boldsymbol{X}(c, \boldsymbol{\gamma})\boldsymbol{\beta} + \boldsymbol{Z}\boldsymbol{u} + \boldsymbol{\epsilon}$$

with parameters $\boldsymbol{\theta} = \{a, b_1, b_2, c, \sigma^2, \tau^2, \nu^2\}$. We further assume $\pi(\nu^2) = \mathrm{IG}(a_\nu, b_\nu)$. The conditional distributions for Gibbs sampler are the same for $\{a, b_1, b_2, c, \sigma^2, \tau^2\}$ except that $\boldsymbol{X}(c)$ is replaced by $\boldsymbol{X}(c, \boldsymbol{\gamma})$, i.e., $x_{ij} - c \vee 0$ replaced by $x_{ij} - c_i \vee 0$, and for updating c the support $[\min_{i,j}(x_{ij}),\, \max_{i,j}(x_{ij})]$ is replaced by

$$[\max_i(\min_j(x_{ij}) - \gamma_i),\ \min_i(\max_j(x_{ij}) - \gamma_i)]$$

and we have

$$\begin{aligned} \pi(\nu^2 \mid \cdots) &= \mathrm{IG}\left(n/2 + a_\nu,\, \boldsymbol{\gamma}'\boldsymbol{\gamma}/2 + b_\nu\right) \\ \pi(\gamma_i \mid \cdots) &\propto \exp\{-\frac{1}{2\sigma^2}\sum_{j=1}^{n_i}(\Delta_{ij} - u_i)^2 - \frac{1}{2\nu^2}\gamma_i^2\} \end{aligned}$$

where $\Delta_{ij} = y_{ij} - a - b_1 (x_{ij} - c - \gamma_i \vee 0) - b_2 (x_{ij} - c - \gamma_i \vee 0)^2$. Again the posterior distribution for γ_i is not a known distribution but can be sampled numerically using Griddy-Gibbs sampler over its support $[\min_j(x_{ij}) - c, \max_j(x_{ij}) + c]$. With the constraint $\sum_i \gamma_i = 0$ we update γ_i for $i = 1, 2, \cdots, n-1$ and let $\gamma_n = -\sum_{i=1}^{n-1} \gamma_i$.

The Mixed-effects Power-Delay Model (MPD) is given by

$$y_{ij} = a + b_2 (x_{ij} - c \vee 0)^d + u_i + \epsilon_{ij}, \quad a, b_2, d \in \mathbb{R}, c \in [\min_{i,j}(x_{ij}), \max_{i,j}(x_{ij})]$$

and the Mixed-effects Power-Delay Model with Varying delays (MPDV)

$$\begin{aligned} y_{ij} &= a + b_2 (x_{ij} - c_i \vee 0)^d + u_i + \epsilon_{ij}, \quad a, b_2, d \in \mathbb{R} \\ c_i &= c + \gamma_i, \quad c_i \in [\min_j(x_{ij}), \max_j(x_{ij})] \end{aligned}$$

Compared to the quadratic models, $\boldsymbol{\beta} = (a, b_1, b_2)'$ is replaced by $\boldsymbol{\beta} = (a, b_2, d)'$ with exponent d. The conditional distributions for common parameters are the same, except $\boldsymbol{X}(c)$ is replaced by $\boldsymbol{X}(c, d)$, i.e., each row $(1,\ x_{ij} - c \vee 0,\ (x_{ij} - c \vee 0)^2)$ is replaced by $(1,\ (x_{ij} - c \vee 0)^d)$ for MPD, and similarly $\boldsymbol{X}(c, \boldsymbol{\gamma})$ is replaced by $\boldsymbol{X}(c, d, \boldsymbol{\gamma})$ for MPDV. For power parameter d, we choose uniform prior over a specified appropriate support that is suggested by the data. Specifically, we choose $\pi(d) = \text{Uniform}(0, 20)$. Similarly the conditional posterior distribution for c is not known and can be computed numerically over the specified range.

References

[1] James GM, Sugar CA: **Clustering for sparsely sampled functional data.** *J Am Stat Assoc* 2003, **98:**397–408.

[2] Ritter C, Tanner MA: **Facilitating the Gibbs sampler: the Gibbs stopper and the Griddy-Gibbs sampler.** *J Am Stat Assoc* 1992, **87:**861–868.
